# Effect of unilateral training and bilateral training on physical performance: A meta-analysis

**DOI:** 10.3389/fphys.2023.1128250

**Published:** 2023-04-13

**Authors:** Wenfeng Zhang, Xing Chen, Kun Xu, Hezhi Xie, Duanying Li, Shicong Ding, Jian Sun

**Affiliations:** Guangzhou Sport University, Guangzhou, China

**Keywords:** unilateral exercises, bilateral exercises, explosive power, maximal force, ability

## Abstract

**Background:** In Unilateral (UNI) exercises are more effective than bilateral (BI) exercises in improving athletic performance is debatable.

**Objectives:** this meta-analysis investigated the effects of UNI and BI exercises on different effect indicators of jump ability, sprint ability, maximal force, change of direction ability, and balance ability.

**Data Sources:** PubMed, Google Scholar, Web of science, CNKI, Proquest, Wan Fang Data.

**Study Eligibility Criteria:** To be eligible for inclusion in the meta-analysis, the study had to be: 1) athletes; 2) UNI training and BI training; 3) the intervention period had to be more than 6 weeks and the intervention frequency had to be more than 2 times/week; 4) the outcome indicators were jumping ability, sprinting ability, maximum strength, and change of direction and balance.

**Study Appraisal and Synthesis Method:** We used the random-effects model for meta-analyses. Effect sizes (standardized mean difference), calculated from measures of horizontally oriented performance, were represented by the standardized mean difference and presented alongside 95% confidence intervals (CI).

**Results:** A total of 28 papers met the inclusion criteria, and Meta-analysis showed that UNI training was more effective than BI training in improving jumping ability (ES = 0.61.0.23 to 0.09; Z = 3.12, *p* = 0.002 < 0.01), sprinting ability (ES = −0.02, −0.03 to −0.01; Z = 2.73, *p* = 0.006 < 0.01), maximum strength (ES = 8.95,2.30 to 15.61; Z = 2.64, *p* = 0.008 > 0.05), change of direction ability (ES = −0.03, −0.06 to 0.00; Z = 1.90, *p* = 0.06 > 0.01) and balance ability (ES = 1.41,-0.62 to 3.44; Z = 1.36, *p* = 0.17 > 0.01). The results of the analysis of moderating variables showed that intervention period, intervention frequency and intervention types all had different indicators of effect on exercise performance.

**Conclusion:** UNI training has a more significant effect on jumping and strength quality for unilateral power patterns, and BI training has a more significant effect on jumping and strength quality for bilateral power patterns.

## 1 Introduction

In his book, scholar Michael Boyle says “While athletes in most sports compete a unilateral pattern of force, many coaches’ training tools are always bilateral” (Boyle, 2003). The design of exercise program is critical for athletes and non-athletes alike, and common variables such as frequency, intensity, and number of sets need to be considered, as well as the selection of training movement patterns.

Recently, UNI exercises such as lunge squats, rear foot elevation split-leg squats, single-leg drop jumps, *etc.*, Have become increasingly popular in physical training programs. As an auxiliary exercise to BI training, UNI training is usually implemented to increase the overall load or to provide training variations (Stone et al., 2007). Specialized characteristics and adaptive migration to the body are important considerations in designing UNI training programs to improve sport performance (Zatsiorsky and Kraemer, 2006). Many studies have shown that the main training method for migrating strength qualities to physical performance is BI training (e.g., squat, deadlift, bench press, *etc.*) (Hoffman et al., 2004; Harris et al., 2008; Comfort et al., 2012). The advantage of BI training is to maximize the use of external loads to develop maximal forces (Stone et al., 2003; Comfort et al., 2012; Seitz et al., 2014). Due to the UNI nature of most characteristics of sports events (e.g., sprinting and change of direction), UNI training is deemed more in line with specific characteristics (Juan, 2001; McCurdy and Conner., 2003). Compared with BI training, UNI training has a smaller range of lower limb support and higher requirements for multi-joint neuromuscular coordination and stability (McCurdy et al., 2010; Makaruk et al., 2011; Jones et al., 2012). Studies have shown that UNI training instability can affect changes in the neuromuscular activation levels of the gluteus medius, hamstring and quadriceps muscles (DeFOREST et al., 2014). However, the unstable support points of UNI training may also limit the strength development of individuals in training and the magnitude of the external load that needs to be applied to subsequently improve athletic performance (Argus et al., 2011).

Currently, there is still some controversy in the academic community regarding the effectiveness and training mechanism of UNI and BI training. There is still much disagreement between the findings that UNI and BI training affect sprinting ability, jumping ability, agility qualities, balance and maximal force. Many sports rely on unilateral movements, such as agility and multidirectional speed in collective ball sports, short distance sprinting, and long jumping, while many sport-specific technical movements are presented in a unilateral form, such as basketball layups, soccer shots, tennis strokes, and golf. Therefore, the specificity of sports efforts is different and the involvement of a specific muscle group will also be different so the effect will be different. No comprehensive systematic evaluation of UNI and BI training on physical performance in different populations has been conducted. Additionally, the effects of UNI and BI training cycles, frequency and duration of intervention on the different effect indicators of physical performance; and whether different testing instruments and methods can accurately evaluate and reflect the subjects’ physical performance are also issues worth studying. Therefore, this study used a systematic review to systematically and objectively evaluate the exact effects of UNI and BI exercises on different effect indicators of athletes’ physical performance from an evidence-based scientific perspective, with the aim of providing a theoretical basis for coaches and athletes.

## 2 Methods

### 2.1 Protocol and registration

The Preferred Reporting Items for Meta-Analyses (PRISMA) was used as the protocol for the design of the review (Altman et al., 2009), and has been registered in the PROSPERO database (protocol number 325983). The PRISMA guidelines include a 27-item checklist considered improving reporting transparency, limits the risk of publication and selection bias (Liberati et al., 2009).

### 2.2 Eligibility criteria

#### 2.2.1 Inclusion criteria

1) Study participants: athletes; 2) Interventions: UNI exercise and BI exercise; 3) The outcome indicators were jumping ability, sprinting ability, maximal force, change of direction ability and balance ability; 4) The sample size, mean and standard deviation were provided.

#### 2.2.2 Exclusion criteria

1) Conference abstracts, review types, *etc.*,; 2) repeatedly published literature with poor quality assessment; 3) literature with data that could not be extracted or combined; 4) experimental participants excluding disabilities or having other physical diseases, *etc.*

### 2.3 Literature search

The databases were searched by 2 researchers each using an independent double-blind approach, and 6 databases were used for the literature search with a search deadline of 20 January 2022 (Table 1).

**TABLE 1:** Literature search criteria settings.

Search items	Content
Data source	PubMed, Google Scholar, Web of science, CNKI, Proquest, Wan Fang
Retrieval format	“Unilateral training” (“Unilateral exercises” OR “Unilateral resistance training” OR “Single leg training” OR “Unilateral limb exercises”)
	“Bilateral training” (“Bilateral exercises” OR “Bilateral resistance training” OR “Bilateral limb exercises”)
	“Jump of ability” (“Jump”)
	“Ability of sprint” (“Sprint”)
	“1 repetition maximum” (”1RM” OR “Squat” OR “Bulgarian spilt squat”)
	“Agility” (“change of direction”)
	“Balance of ability” (“balance” OR “dynamic balance”)
	“Athletes (“players”)
Language of literature	Unlimited
Type of literature	Journal, Thesis
Search date	1 January 2011 ∼ 20 January 2022

### 2.4 Study selection

Two authors independently assessed the suitability of the titles and abstracts of the search results. If the title or abstract met the eligibility criteria or there was uncertainty, the full-text article was retrieved. In case of disagreement, a third author was consulted. Also, the reasons for excluding articles are recorded.

### 2.5 Data collection processes

A data collection form was created using the Cochrane Data Extraction and Evaluation Form template. One author was responsible for collecting the data and a second author was responsible for checking the extracted data. In case of disagreement, a third author was consulted.

### 2.6 Data items

Two personnel used an independent double-blind approach during the search process to extract relevant indicators from the included literature, including the first author, year, gender, population, program, training period, and frequency of intervention (times/week).

### 2.7 Risk of bias of individual studies

The Physiotherapy Evidence Database (PEDro) was used to assess the risk of bias and methodological quality of studies included in the meta-analysis, and the scale assessed the validity of studies on a scale from 0 (high risk of bias) to 10 (low risk of bias). The scale was evaluated by three persons independently for the included studies, and if the evaluations differed, they met to discuss. The first item was not counted in the total score, and a total score ≥6 represented a low risk of bias threshold and high quality of the literature.

### 2.8 Summary of measures

The primary outcomes assessed in this meta-analysis are jump performance, sprint performance, Maximal force, change of direction ability, balance performance.

### 2.9 Synthesis of methods

Effect size merging, subgroup analysis, and heterogeneity testing was performed by Review Manger 5.0 statistical software, and because the outcome indicators of the included literature were continuous variables, the effect scale indicators were selected as mean difference (MD) and 95% confidence intervals. The I^2^ statistic was used to evaluate the heterogeneity of all included literature. When 0 ≤ I^2^ < 25, it indicated no heterogeneity among studies; 25 ≤ I^2^ < 50, mild heterogeneity existed; 50 ≤ I^2^ < 75, moderate heterogeneity existed; I^2^ ≥ 75%, severe heterogeneity existed. If I^2^ > 50, the method for changing the effect model was chosen to assess the sensitivity of this Meta-analysis, and the changes in RR (OR) and MD (SMD) were observed after changing the effect model.

### 2.10 Risk of bias across all studies

Publication bias was quantified by Stata SE12.0 software Egger’s test, *p* < 0.05 significant publication bias.

## 3 Results

### 3.1 Study selection

**FIGURE 1:**
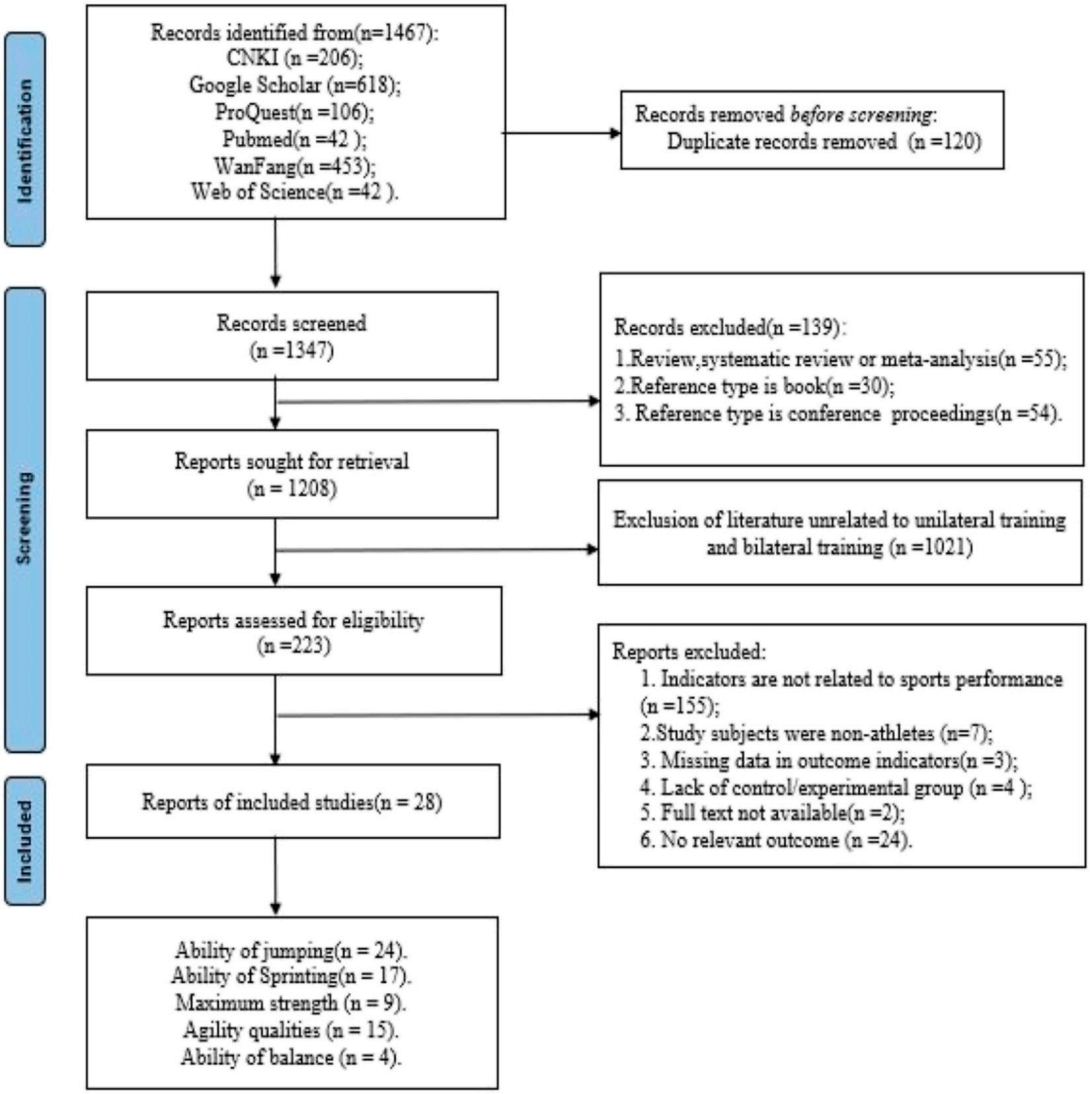
Flowchart for inclusion and exclusion of studies.

A preliminary search of 1,467 literature was conducted and after excluding repetitive literature, CNKI (n = 197), Google Scholar (n = 611), Proquest (n = 77), PubMed (n = 7), WanFang Data (n = 437), Web of science (n = 15). A total of 28 studies met the inclusion criteria and were included in the meta-analysis (Figure 1).

### 3.2 Study characteristics

After screening and reading, a total of 28 papers met the inclusion criteria. Of these, 327 athletes completed UNI training and 324 athletes completed BI training. The training period was 6–12 weeks and the training frequency was 2–3 times per week. The main types of interventions for UNI and BI training were resistance training, rapid stretch complex training and compound training (Table 2).

**TABLE 2:** List of basic characteristics of the included literature Abbreviations: M = male; F = female; RT = Resistance training; PT = Plyometric training; CT = complex training; EOT = Eccentric-overload training.

Study		Participants						Training program						Outcome measure
Author, Year	nation	Sex	Identity	N	Age	Height	Weight	Weeks	Times/week	Type	Exercises	Sets	Reps	Performance
Fisher and Wallin (2014)	Britain	M	Rugby players	UNI(8)	20.14 ± 1.77	180.00 ± 6.00	85.70 ± 7.06	6	2	CT	Single Leg Squat, Forward Hop, Lateral Hop, Hexagon Hop*etc.*	1–3	6–10	Sprint; Change of direction ability
				BI(7)	19.80 ± 1.49	182.00 ± 8.00	82.60 ± 6.52				Back Squat, Forward Jump, Lateral Jump*etc.*	1–3	6–10	
Mudlo (2014)	United States	5F,6M	Swimming players	UNI(11)	20.45 ± 1.13	—	—	8	2	CT	single leg squat, single leg push off, split squat jump*etc.*	30s–1.5min	5–15	Jump; Change of direction ability
		5F,5M		BI(11)	20.30 ± 1.30	—	—				bilateral back squat, double leg vertical jump, double leg vertical jump with a leg tuck*etc.*	30s∼3min	5–15	
Peng (2016)	China	M	Wrestling players	UNI(6)	17.00 ± 2.00	178.30 ± 5.15	78.83 ± 5.24	8	8	RT	Weighted Single Leg Squat	6	5	Jump; Maximum force
				BI(6)	17.50 ± 1.50	172.30 ± 6.51	83.50 ± 5.3				Weighted Squat	6	5	
Elliott (2016)	Sweden	F	Handball players	UNI(12)	20.30 ± 2.30	174.00 ± 5.71	71.60 ± 7.60	16	2	RT	Marklyft Enbens, Enbens benböj, Enbens benböj knix*etc.*	2–4	3–10	Jump; Change of direction ability; Balance
				BI(7)	19.90 ± 1.60	174.60 ± 6.99	70.80 ± 7.81				Marklyft, Benböj halva, Benböj knix*etc.*	2–4	3–10	
Speirs et al. (2016)	Britain	M	Rugby players	UNI(9)	18.10 ± 0.50	183.00 ± 3.40	96.70 ± 9.30	5	2	RT	Rear elevated split squat (RESS)	4	3–6	Sprint; Maximum force; Change of direction ability
				BI(9)	18.10 ± 0.50	185.00 ± 8.90	98.10 ± 13.40				Back squat	4	3–6	
Zhao (2017)	China	F	Basketball players	UNI(7)	U15	177.92 ± 8.64	68.85 ± 9.84	8	3	CT	Single Leg Jump Deep, Bulgarian Cut Squat, Barbell single-leg hard pull.etc	3–5	3–15	Jump
				BI(10)							Double-legged deep jump, Barbell Back Neck Squat, Barbell hard pull*etc.*	3–5	6–8	
Potter (2017)	United States	M,F	Soccer players	UNI(18)	19.61 ± 1.29	176.00 ± 9.00	71.59 ± 7.60	6	3 times in the first 3 weeks,2 times in the last 3 weeks	RT	Barbell Single Leg RDLs, Step-Ups, Bulgarian Split Squats	—	—	Jump; Change of direction ability
				BI(16)	20.00 ± 1.15	176.00 ± 8.00	68.83 ± 7.92				Trap Bar Deadlifts, Glute Bridge, Hip Thrusts, Barbell Front Squats	—	—	
Gonzalo-Skok, et al. (2017a)	Spain	M	Basketball players	UNI(9)	16.80 ± 1.70	190.40 ± 6.90	76.90 ± 8.60	6	2	RT	90°-squat, drop jumps (25 cm), CMJ	2–3	5	Jump; Sprint; Change of direction ability
				BI(9)	16.70 ± 1.70	188.90 ± 7.50	74.90 ± 9.60				90°-squat, drop jumps (50 cm), CMJ	2–3	5	
Gonzalo-Skok et al. (2017b)	Spain	M	Amateur athletes	UNI(24)	20.50 ± 2.00	180.10 ± 6.30	73.20 ± 9.30	8	2	RT (EOT)	Variable Unilateral Multidirectional	1	6–10	Jump; Sprint; Change of direction ability
				BI(24)							Constant Bilateral Vertical	1	6	
Vaughan (2018)	United States	F	Field hockey players	UNI(10)	18–21	—	—	6	2	CT	1 Leg Hurdle Hop, 1 Leg Standing Long Jump, 1 Leg Hang Power Clean, 1 Leg Loaded Squat Jump*etc.*	3–6	—	Jump
				BI(9)							Hurdle Jump, Standing Long Jump, Hang Power Clean, Loaded Squat Jump, Barbell RDL*etc.*	3–6	—	
Ramirez-Campillo et al. (2018)	Chile	M	Soccer players	UNI(9)	17.30 ± 1.10	177.10 ± 5.90	64.90 ± 5.50	8	2	CT	Knee extensors, Knee flexors, horizontal drop jumps, Horizontal jumps	1–3	3–10	Jump; Change of direction ability
				BI(9)	17.60 ± 0.50	174.90 ± 5.30	68.30 ± 3.60				Knee extensors, Knee flexors, horizontal drop jump, Horizontal jumps	1–3	3–10	
Qichao (2018)	China	M	Basketball players	UNI(6)	21.00 ± 0.894	188.17 ± 4.54	87.77 ± 4.49	10	3	CT	30 m timed single-leg jump, Single Leg Step Jump*etc.*	3	10	Jump; Sprint
				BI(6)	21.00 ± 1.265	188.83 ± 3.97	87.77 ± 4.49				Double-legged jumping bar frame, Jumping steps with both legs, Barbell lunge for leg jump barbell*etc.*	3	6–10	
Basilios (2018)	Greece	M	Soccer players	UNI(23)	9.94 ± 1.80	142.22 ± 8.66	39.29 ± 8.18	10	2	PT	Horizontal jump, Continuous jumping*etc.*	3–5	6–10	Jump; Sprint; Change of direction ability
				BI(23)	9.95 ± 1.47	139.15 ± 7.03	36.12 ± 7.82				Horizontal jum (Z., 2018)p, Continuous jumping*etc.*	3–5	6–10	
Ye and Wangcheng (2018)	China	M	Track and field players	UNI(6)		177.00 ± 9.00	76.92 ± 6.06	8	3	RT	single Leg suspension power cleaning, Single leg sit-up	6–8	2–6	Jump; Sprint
				BI(6)		179.00 ± 12.00	77.14 ± 5.19				Double leg suspension power cleaning, Double leg sit-up	6–8	2–6	
Gonzalo-Skok et al. (2019)	Spain	M	Basketball players	UNI(9)	13.20 ± 0.50	171.70 ± 7.20	59.60 ± 11.70	6	2	PT (EOT)	unilateral-horizontal, Drop Jump 10cm, SLJ, SLJ without CMJ*etc.*	2–5	2–5	Jump; Sprint; Change of direction ability
				BI(9)	13.00 ± 0.60	172.80 ± 7.90	59.10 ± 1.80				bilateral-vertical, Drop Jump 20cm, SJ with arms swing*etc.*	2–5	2–5	
Dongfeng (2019)	China	M	Track and field players	UNI(8)	19.88 ± 0.64	177.50 ± 3.20	69.13 ± 4.08	8	2	CT	Bulgarian Lunge Squat + Single leg jump deep max long jump	4	3–10 + 6–8	Jump; Sprint
				BI(9)	20.11 ± 0.78	178.67 ± 4.52	69.56 ± 9.67				Half Squat + Double leg jump deep max jump	4	3–10 + 3–4	
Shaosong (2019)	China	M	Basketball players	UNI(10)		184.50 ± 5.50	83.50 ± 5.60	6	2	RT	Single leg hard pull	5	5times/side	Jump; Maximum force
				BI(10)		183.90 ± 3.70	82.80 ± 10.00				Double leg hard pull	5	5	
Yan and Hao (2019)	China	F	Judo players	UNI(8)	20.5	160.60 ± 3.40	50.82 ± 7.64	10	3	RT	Single leg back extension squat, Single Leg Lateral Extension Squat, Single leg front extension squat	3	8–12	Jump; Maximum force; Balance
				BI(8)							Left and right split-leg squats, Front and back split-leg squats, Weighted Back Neck Squat*etc.*	2–5	8–24	
Appleby, et al. (2020)	Australia	M	Rugby players	UNI(10)	23.10 ± 4.10	186.30 ± 5.10	104.60 ± 11.50	12	2	RT	Step-up	6–8	4–8	Maximum force
				BI(13)	21.80 ± 3.30	184.30 ± 5.90	101.30 ± 12.80				Squat	6–8	4–8	
Abston (2020)	United States	M,F	Weightlifting players	UNI(7)	18–25	—	—	6	3	PT	SJ, CMJ, Depth Drop	4	6–8	Sprint
				BI(7)								4	3–4	
Yilin (2020)	China	M	Soccer players	UNI(7)	—	—	—	6	3	CT	Rear leg squat, Single leg 20 cm jump depth, Single Leg Vertical Jump			Jump; Change of direction ability
				BI(5)	—	—	—				Barbell Half Squat, Double-legged 40 cm jumping depth*etc.*			
Boxuan (2020)	China	M	Ice hockey players	UNI(7)	15.75 ± 0.66	174.67 ± 3.94	64.56 ± 4.67	8	2	RT	Bulgarian Squat	2	4	Jump; Sprint; Balance
				BI(7)	15.625 ± 0.99	171.71 ± 2.49	62.78 ± 10.85				Weighted Squat	2	8	
Stern et al. (2020)	Britain	M	Soccer players	UNI(11)	17.60 ± 1.20	179.66 ± 7.27	77.30 ± 7.91	6	2	CT	Rear foot elevated split squat, Single-leg drop jump, Single-leg countermovement jump*etc.*	5	6	Jump; Sprint; Maximum force; Change of direction ability
				BI(12)							Back squat, Drop jump, Countermovement jump, Broad jumps*etc.*	5	6	
(Drouzas et al., 2020)	Greece	M	Soccer players	UNI(23)	9.90 ± 1.80	142.20 ± 8.70	39.30 ± 8.20	10	2	PT	Jumps in nine squares, Jumps over hurdles, Jumps in four directions after light signal*etc.*	4	3–6	Jump; Sprint; Change of direction ability
				BI(23)	10.00 ± 0.50	139.20 ± 7.00	36.10 ± 7.80					4	3–6	
Ahmad and Jain (2020)	India	M	Volleyball players	UNI(33)	16.16 ± 1.65	167.14 ± 6.57	59.51 ± 9.03	8	2	PT	—	2–5	3–6	Jump
				BI(33)	16.18 ± 1.80	164.07 ± 2.34	55.80 ± 4.36				—	3–5	6–10	
Fahui (2021)	China	M	Soccer players	UNI(14)	U18	176.40 ± 2.80	69.60 ± 2.80	8	3	CT	Bulgarian Squat, Rear leg raise split leg squat jump, Single Leg Romanian Hard Pull, Single leg continuous jump	5	5	Jump; Sprint; Maximum force; Change of direction ability
				BI(14)		178.10 ± 2.90	70.10 ± 4.30				Squat, squat jump, Romanian hard pull, Continuous jumping with both legs	5	5	
Yibiao (2021)	China	M	Basketball players	UNI(12)	20.81 ± 1.06	182.89 ± 7.67	75.38 ± 11.92	6	3	CT	Lunge Squat + Single leg push stirrup on box	3	4∼5times + 5times/side	Jump; Sprint; Maximum force
				BI(12)	21.76 ± 1.64	184.67 ± 8.44	77.58 ± 11.10				Squat + Jump box	3	4∼5times + 5times	
Sufan (2021)	China	F	Basketball players	UNI(10)	16.30 ± 0.67	175.00 ± 6.96	67.80 ± 10.50	12	3	CT	Bulgarian Lunge Squat, Single leg vertical jump, Single Leg Continuous Long Jump*etc.*	5	5–10	Jump; Sprint; Maximum force; Change of direction ability
				BI(10)	16.20 ± 0.78	174.00 ± 8.23	73.30 ± 13.40				Barbell weighted half squat, Vertical jump with both legs, Double leg continuous long jump*etc.*	5	5–11	

Abbreviations: M = male; F = female; RT, resistance training; PT, plyometric training; CT, complex training; EOT, Eccentric-overload training.

### 3.3 Risk of bias within studies

There were 23 literature quality scores ≥6 as assessed by the PEDro scale (Table 3).

**TABLE 3:** The Physiotherapy Evidence Database (PEDro) scale ratings.

Authors, year	N1	N2	N3	N4	N5	N6	N7	N8	N9	N10	N11	Total
Fisher and Walin (2014)	0	1	1	1	0	0	0	1	1	1	1	7
Mudlo (2014)	1	1	1	0	0	0	0	1	1	1	1	6
Peng (2016)	1	1	0	1	0	0	0	1	1	1	1	6
(Elliott, 2016)	0	1	0	0	0	0	0	1	1	1	1	4
Speirs et al. (2016)	1	1	1	1	0	0	0	1	1	1	1	7
Zhao (2017)	1	1	0	1	0	0	0	1	1	1	1	6
Potter (2017)	0	1	0	1	0	0	0	1	1	1	1	6
Gonzalo-Skok et al. (2017a)	0	1	0	1	0	0	0	1	1	1	1	6
Gonzalo-Skok et al. (2017b)	1	1	0	1	0	0	0	1	1	1	1	6
Ramirez-Campillo et al. (2018)	0	1	1	1	0	0	0	1	1	1	1	7
Qichao (2018)	0	1	1	1	0	0	0	1	1	1	1	7
Basilios (2018)	1	0	0	1	0	0	0	1	1	1	1	5
Ye and Wangcheng (2018)	0	1	1	1	0	0	0	1	1	1	1	7
Gonzalo-Skok, et al. (2019)	0	1	0	1	0	0	0	1	1	1	1	6
Dongfeng (2019)	1	0	0	1	0	0	0	1	1	1	1	5
Shaosong (2019)	1	1	1	1	0	0	0	1	1	1	1	7
Yan and Hao (2019)	0	0	0	1	0	0	0	1	1	1	1	5
Appleby et al. (2020)	1	1	1	0	0	0	0	1	1	1	1	6
Abston (2020)	0	1	1	1	0	0	0	1	1	1	1	7
Yinlin (2010)	1	1	0	1	0	0	0	1	1	1	1	6
Boxuan (2020)	0	1	1	0	0	0	0	1	1	1	1	6
Stern et al. (2020)	1	1	0	1	0	0	0	1	1	1	1	6
Drouzas et al. (2020)	1	1	1	1	0	0	0	1	1	1	1	7
Ahmad and Jain (2020)	0	1	0	1	0	0	0	1	1	1	1	6
Fahui (2021)	1	1	1	1	0	0	0	1	1	1	1	7
Yibiao (2021)	1	1	1	1	0	0	0	1	1	1	1	7
Sufan (2021)	0	0	0	1	0	0	0	1	1	1	1	5

Abbreviations: N1 = inclusion criteria; N2 = Random-ization; N3 = concealed allocation; N4 = baseline comparison; N5 = blind participants; N6 = blind therapysts; N7 = blind assessors; N8 = Adequate follow-up; N9 = Intention-to-treat analysis; N10 = Between group comparisons; N11 = Point estimates and variability.

### 3.4 Results of individual studies

#### 3.4.1 Maximal force

**FIGURE 2:**
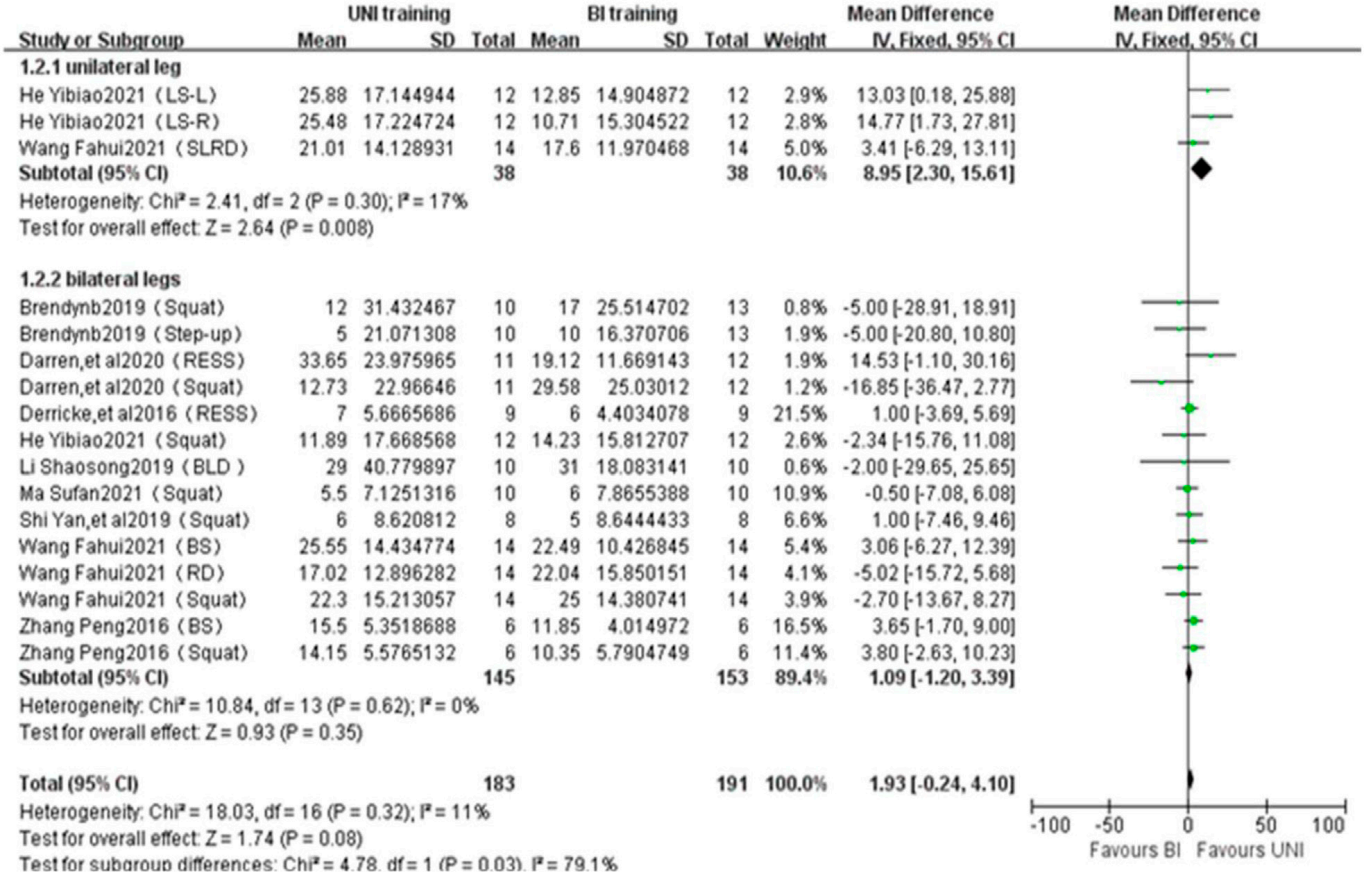
Intergroup Forest plots of UNI and BI training affecting Maximal Force. RESS = rear elevated split squat; LS-L = lunge squat with left leg; LS-R = lunge squat with right leg; BLD = bended-leg deadlift; BS = Bulgarian Squat; RD = Romanion deadlift; SLRD = Single-Leg Romanion deadlift.

A total of 17 studies from 9 publications were included to report the effects of UNI and BI training on maximal force (Figure 2). The statistical difference between UNI on single-leg maximum strength (ES = 8.95,2.30 to 15.61; Z = 2.64, *p* = 0.008 < 0.01), with no heterogeneity between studies (I^2^ = 17%, *p* = 0.30); UNI did not differ statistically for maximum strength in both legs (ES = 1.09, −1.20 to 3.39; Z = 0.93, *p* = 0.35 > 0.05), and there was no heterogeneity between studies (I^2^ = 0%, *p* = 0.62). Maximum force was measured in kilograms (kg).

#### 3.4.2 Jump performance

**FIGURE 3:**
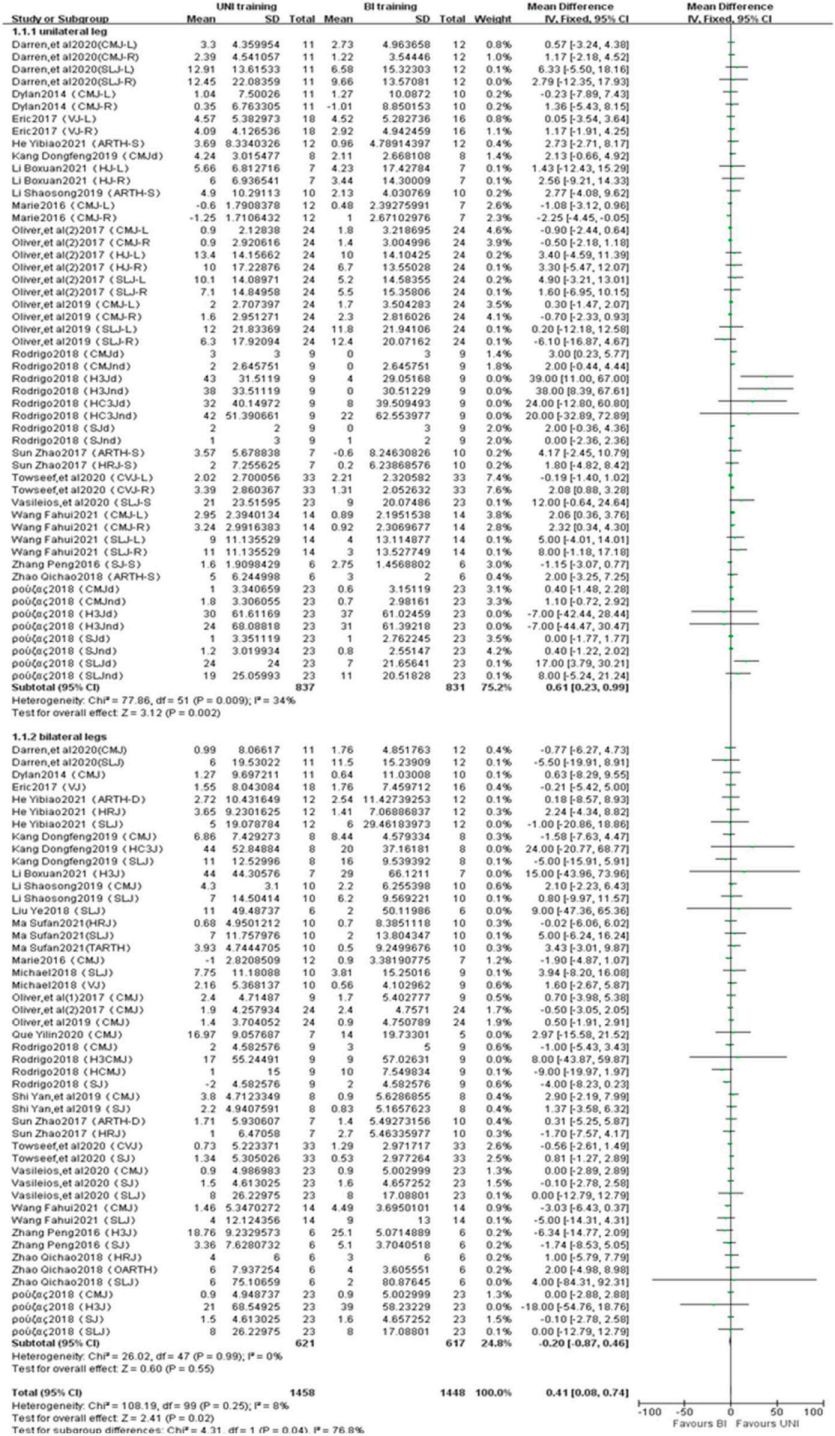
Intergroup Forest plots of UNI and BI training affecting jumping ability. CMJ = countermovement jump; CMJ-L = countermovement jump with left leg; CMJ-R = countermovement jump with right leg; CMJd = countermovement jump with dominant leg; CMJnd = countermovement jump with non-dominant leg; CMJ-S = countermovement jump with single leg; SLJ = standing long jump; SLJ-L = standing long jump with left leg; SLJ-R = standing long jump with right leg; SLJd = standing long jump with dominant leg; SLJnd = standing long jump with non-dominant leg; SLJ-S = standing long jump with single leg; VJ = vertical jump; VJ-L = vertical jump with left leg; VJ-R = vertical jump with right leg; VJd = vertical jump with dominant leg; ARTH-D = assisted running double feet touch high; ARTH-S = assisted running single foot touch high; HRJ = highest reach jump; HRJ-S = highest reach jump with single leg; HC3J = cross and horizontal triple jump; H3J = horizontal triple jump; H3J-R = horizontal triple jump with right leg; H3Jd = horizontal triple jump with dominant leg; H3Jnd = horizontal triple jump with non-dominant leg; HJ-L = horizontal jump with left leg; HJ-R = horizontal jump with right leg; TARTH = three-step assisted running touch high; H3CMJ = triple bilateral horizontal jump with arm swing; HCMJ = bilateral horizontal jump with arm swing; CVJ = countermovement vertical jump; CVJ-L = countermovement vertical jump with left leg; CVJ-R = countermovement vertical jump with right leg; SJ = squat jump; SJd = squat jump with dominant leg; SJnd = squat jump with non-dominant leg; OARTH = one-step assisted running touch high.

A total of 100 studies from 24 publications were included to report the effects of UNI and BI training on jumping ability (Figure 3). The statistical difference in UNI on single-leg jumping ability (ES = 0.61,0.23 to 0.09; Z = 3.12, *p* = 0.002 < 0.01) with mild heterogeneity between studies (I^2^ = 34%, *p* = 0.009); UNI did not differ statistically for jumping ability on both legs (ES = −0.20,-0.87 to 0.46; Z = 0.60, *p* = 0.55 > 0.05), and there was no heterogeneity between studies (I^2^ = 0%, *p* = 0.99). Jump performance was measured in centimeters (cm).

#### 3.4.3 Linear sprint performance

**FIGURE 4:**
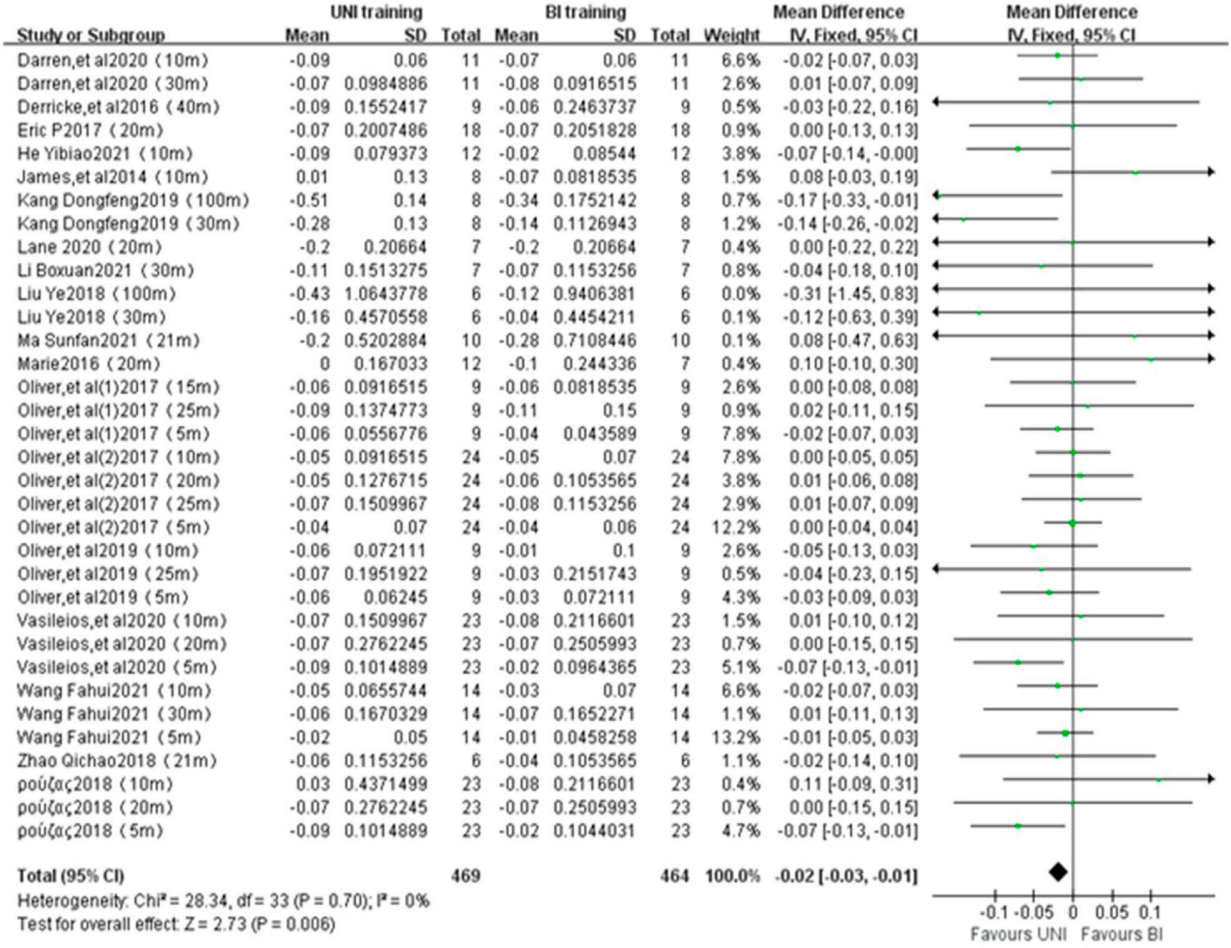
Intergroup Forest plots of UNI and BI training affecting sprinting ability.

A total of 34 studies from 17 publications were included to report the effect of UNI and BI training on linear sprint performance (Figure 4). The statistical differences were observed (ES = −0.02, −0.03 to −0.01; Z = 2.73, *p* = 0.006 < 0.01). There was no heterogeneity between the studies (I^2^ = 0%, *p* = 0.70). The sprint performance was measured in seconds(s).

#### 3.4.4 Change of direction ability

**FIGURE 5:**
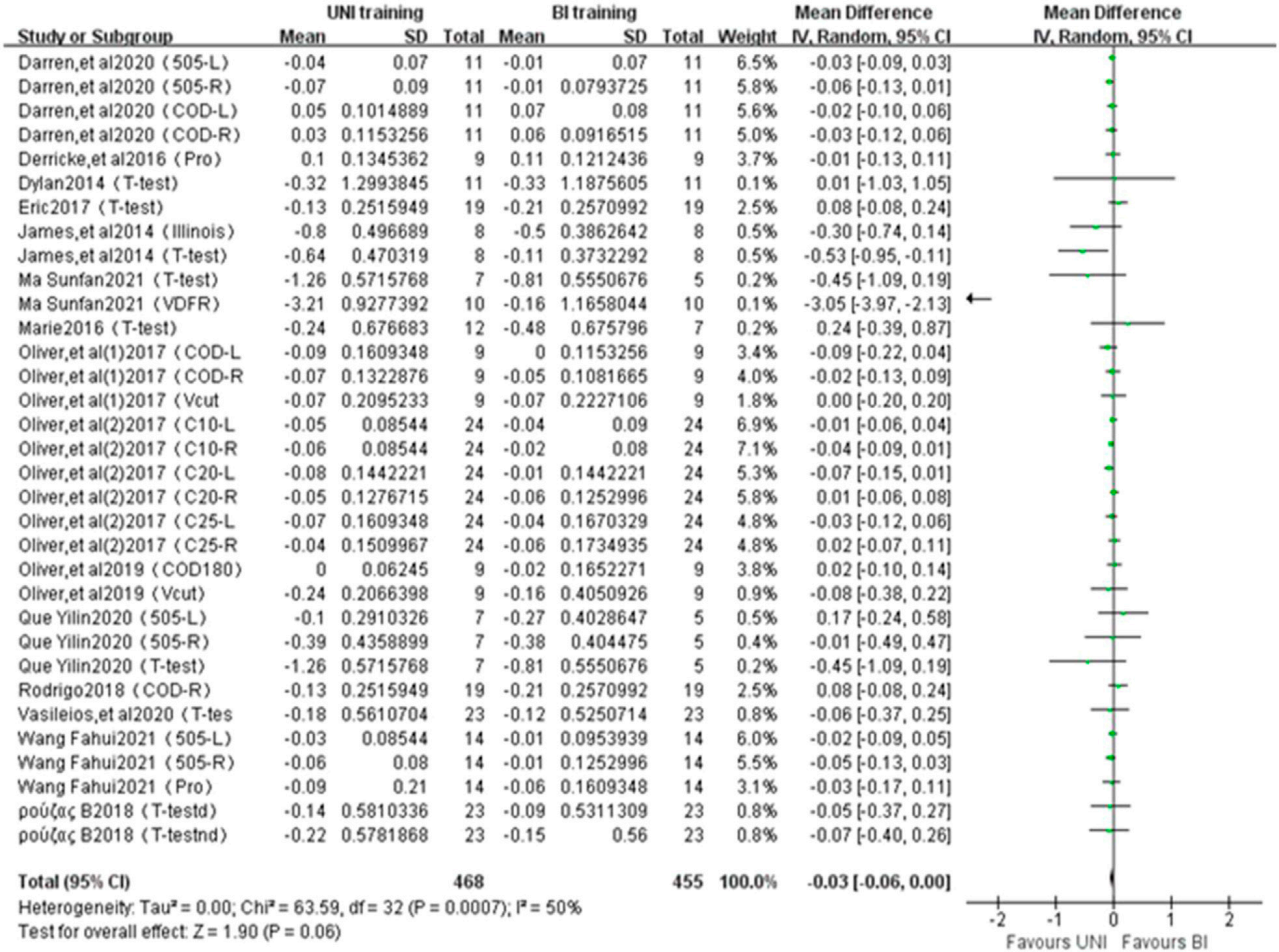
Intergroup Forest plots of UNI and BI training affecting Change of Direction Ability. 505-L = 505 change-of-direction speed test with left leg; 505-R = 505 change of direction speed test with right leg; COD = change-of-direction test; Pro = pro-agility test; COD180 = time in 5 + 5 m sprint change of direction of 180°; COD180d = time in 5 + 5 m sprint change of direction of 180° with dominant leg; COD180d = time in 5 + 5 m sprint change of direction of 180° with non-dominant leg; COD90d = time in 5 + 5 m sprint change of direction of 90° with dominant leg; COD90d = time in 5 + 5 m sprint change of direction of 90° with non-dominant leg; COD-L = change-of-direction test with left leg; COD-R = change-of-direction test with right leg; Illinois = Illinois test; V-cut = 25-m sprint with 4 changes of direction of 45°; C10-L = 10 m with left leg with a COD of 180°; C10-R = 10 m with right leg with a COD of 180°; C20-L = 20 m with left leg with a COD of 180°; C20-R = 20 m with right leg with a COD of 180°; C25-L = 25 m with left leg with a COD of 180°; C25-R = 25 m with right leg with a COD of 180°; *t*-test = T-figure route agility test; *t*-test = T-figure route agility test with dominant leg; *t*-test = T-figure route agility test with non-dominant leg.

A total of 33 studies from 15 publications were included to report the effect of UNI and BI training on change of direction ability (Figure 5). The statistical differences were observed (ES = −0.03, −0.06 to 0.00; Z = 1.90, *p* = 0.06 > 0.01). Moderate heterogeneity between studies existed (I^2^ = 50%, *p* = 0.0007). The change of direction ability was measured in seconds(s).

#### 3.4.5 Balance performance

**FIGURE 6:**
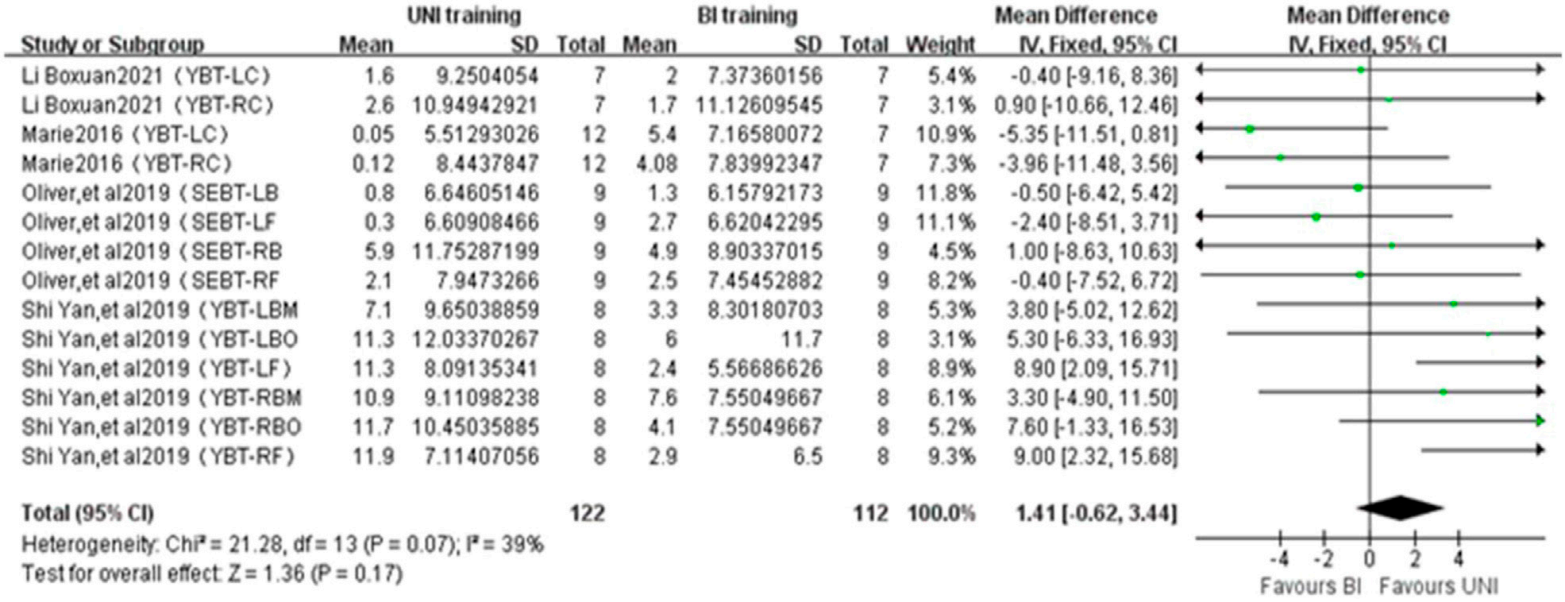
Intergroup Forest plots of UNI and BI training affecting Balance Performance. YBT-LC = Y-Balance test of left comprehensive; YBT-RC = Y-Balance test of right comprehensive; YBT-LBM = Y-Balance test of left back middle; YBT-LBO = Y-Balance test of left back outer; YBT-LF = Y-Balance test of left front; YBT-RBM = Y-Balance test of right back middle; YBT-RBO = Y-Balance test of right back outer; YBT-RF = Y-Balance test of right front; SEBT-LB = star excursion balance test in the left posterior-lateral direction; SEBT-LF = star excursion balance test in the left anterior direction; SEBT-RB = star excursion balance test in the right posterior-lateral direction; SEBT-RF = star excursion balance test in the right anterior direction.

A total of 14 studies from 4 publications were included to report the effect of UNI and BI training on change of direction ability (Figure 6). The statistical differences were observed (ES = 1.41,-0.62 to 3.44; Z = 1.36, *p* = 0.17 > 0.01). Mild heterogeneity was observed between studies (I^2^ = 26%, *p* = 0.14). The balance performance was measured in centimeters (cm).

### 3.5 Results of syntheses

Through sensitivity analysis, it was found that the combined effect values under different effect models were close, indicating that the results of this meta-analysis were stable and reliable.

### 3.6 Effect of moderator variables

A moderating variable is any variable included in the Meta-analysis that helps explain more of the methodological differences (Xiaoyu et al., 2018). To further explore the effect of UNI, BI training on exercise performance, a random effects model was therefore used to group the interventions according to their periodicity, frequency and type, according to literature distribution characteristics. The specific analysis results are shown in Table 4 (UNI) and Table 5 (BI).

**TABLE 4:** Study of the effect of UNI training on physical performance.

Varia-bles	No.of studies					Effectsize [95% CI]					P					I^2^ (%)				
	JP	SP	MP	CODA	BP	MP	JP	SP	CODA	BP	MP	JP	SP	CO DA	BP	MP	JP	SP	CODA	BP
Total	17	100	34	33	14	15.39 [11.48,19.30]	2.52 [1.94,3.09]	−0.08 [-0.10,-0.05]	−0.10 [-0.14,-0.05]	4.77 [2.03,7.50]	<0.01	<0.01	<0.01	<0.01	<0.01	57	48	52	79	42
Trainingcycle																				
≥8	10	75	23	17	10	14.40 [9.96,18.85]	2.29 [1.64,2.95]	−0.09 [-0.12,-0.05]	−0.11 [-0.18,-0.05]	6.35 [2.65,10.05]	<0.01	<0.01	<0.01	<0.01	<0.01	56	53	67	82	52
<8	7	25	11	16	4	18.73 [9.75,27.70]	3.20 [2.28,4.12]	−0.07 [-0.09,-0.04]	−0.09 [-0.15,-0.02]	1.47 [-2.04,4.97]	<0.01	<0.01	<0.01	0.01	= 0.41	64	0	0	75	0
Interve-ntionfreque-ncy																				
>2	9	25	8	8	6	17.02 [10.94,23.10]	3.37 [2.41,4.33]	−0.04 [-0.07,-0.01]	−0.47 [-0.69,-0.26]	10.84 [7.27,14.42]	<0.01	<0.01	<0.01	<0.01	<0.01	68	0	0	93	0
≤2	8	72	25	24	8	13.19 [8.26,18.13]	2.20 [1.54,2.86]	−0.08 [-0.11,-0.06]	−0.05 [-0.08,-0.02]	0.93 [-1.46,3.32]	<0.01	<0.01	<0.01	<0.01	= 0.45	40	53	60	46	0
Mix		3	1	1			3.88 [1.91,5.86]	−0.07 [-0.20,0.06]	−0.12 [-0.50,0.26]			<0.01	= 0.29	= 0.53			0	NA	NA	
Interventiontype																				
RT	7	23	13	16	10	10.96 [6.93,14.99]	2.49 [1.24,3.73]	−0.05 [-0.08,-0.03]	−0.04 [-0.06,-0.02]	6.35 [2.65,10.05]	<0.01	<0.01	<0.01	<0.01	<0.01	31	66	0	6	52
PT	0	28	10	3	4		2.00 [1.16,2.84]	−0.07 [-0.10,-0.05]	−0.11 [-0.30,0.07]	1.47 [-2.04,4.97]		<0.01	<0.01	0.23	= 0.41		46	0	70	0
CT	10	49	11	14		19.16 [13.18,25.15]	3.01 [2.18,3.83]	−0.11 [-0.17,-0.05]	−0.42 [-0.59,-0.25]		<0.01	<0.01	<0.01	<0.01		61	18	84	89	

Abbreviations:JP , Jump performance; SP , sprint performance; MP , maximum force; CODA , change of direction ability; BP , balance performance; RT , resistance training; PT , plyometric training; CT , complex training; NA, Only one literature could not be tested for heterogeneity. The presence of "-" in front of the analyzed values indicates the improvement of the performance in the sprint category.

**TABLE 5:** Study of the effect of BI training on physical performance.

Varia-bles	No.ofstudies						Effectsize [95% CI]						P						I^2^ (%)				
	JP	SP	MP	CODA	BP		MP	JP	SP	CODA	BP		MP	JP	SP	CO DA	BP		MP	JP	SP	CODA	BP
Total	100	34	17	33	14		14.38 [10.80,17.96]	2.16 [1.65,2.68]	−0.05 [-0.06,-0.03]	−0.02 [-0.04,0.00]			<0.01	<0.01	<0.01	0.10	<0.01		63	43	0	28	0
Trainingcycle																							
≥8	75	23	10	17	10		13.96 [9.66,18.25]	2.19 [1.57,2.81]	−0.05 [-0.07,-0.03]	−0.04 [-0.06,-0.01]	3.81 [1.41,6.20]		<0.01	<0.01	<0.01	<0.01	<0.01		61	54	23	0	0
<8	25	11	7	16	4		15.77 [8.64,22.90]	2.25 [1.33,3.16]	−0.05 [-0.07,-0.03]	−0.01 [-0.05,0.04]	2.53 [-0.71,5.78]		<0.01	<0.01	<0.01	= 0.80	= 0.13		68	0	0	50	0
Interventionfrequency																							
>2	25	8	9	8	6		14.81 [9.53,20.08]	1.81 [1.01,2.62]	−0.02 [-0.05,0.00]	−0.05 [-0.13,0.02]	3.92 [1.01,6.84]		<0.01	<0.01	0.09	= 0.17	<0.01		63	0	0	28	0
≤2	72	25	8	24	8		13.78 [8.74,18.81]	2.23 [1.58,2.88]	−0.05 [-0.07,-0.04]	−0.02 [-0.04,0.01]	2.92 [0.36,5.48]		<0.01	<0.01	<0.01	= 0.23	= 0.03		64	55	2	32	0
Mix	3	1		1				3.30 [1.06,5.55]	−0.07 [-0.20,0.06]	−0.06 [-0.51,0.39]				= 0.004	= 0.31	= 0.79				0	NA	NA	
Interventiontype																							
RT	23	13	7	16	10		10.29 [6.03,14.56]	1.89 [1.13,2.66]	−0.05 [-0.07,-0.03]	−0.01 [-0.03,0.02]	3.81 [1.41,6.20]		<0.01	<0.01	<0.01	= 0.54	<0.01		53	0	0	34	0
PT	28	10	0	3	4			2.48 [1.42,3.54]	−0.03 [-0.06,-0.01]	−0.05 [-0.18,0.07]	2.53 [-0.71,5.78]		<0.01	<0.01	0.02	= 0.41	= 0.13			74	0	0	0
CT	49	11	10	14			17.17 [12.53,21.81]	1.98 [1.30,2.67]	−0.07 [-0.10,-0.03]	−0.09 [-0.16,-0.02]			<0.01	<0.01	<0.01	= 0.01			51	13	59	28	

Abbreviations:JP , Jump performance; SP , sprint performance; MP , maximum force; CODA , change of direction ability; BP , balance performance; RT , resistance training; PT , plyometric training; CT , complex training; NA, Only one literature could not be tested for heterogeneity. The presence of "-" in front of the analyzed values indicates the improvement of the performance in the sprint categ.

### 3.7 Sensitivity analysis

Sensitivity analyses were performed by changing the criteria for inclusion selection, the statistical model, and the selection of effect sizes by performing sensitivity analyses on the exercise performance of different effect indicators, and re-running the Meta-analysis, and no significant changes were found in the final evaluation results.

### 3.8 Risk of bias across studies

Bias analysis was performed using the Egger test of Stata SE12.0 to more accurately evaluate the possible publication bias in the study in a combined qualitative and quantitative manner. The results showed no significant publication bias for jumping ability (*p* = 0.463), sprinting ability (*p* = 0.198), maximum strength (*p* = 0.163) and change of direction ability (*p* = 0.021). However, there was a significant publication bias in equilibrium capacity (*p* = 0.007). (Table 6).

**TABLE 6:** Egger’s test results.

Indicators	Std_Eff	Coef	Std.Err	t	P>|t|	[95% Conf.Interval]	
Jump performance	slope	0.0083881	0.146,399	0.06	0.954	−0.282,136	0.2,989,122
	bias	0.2,872,471	0.3,901,455	0.74	0.463	−0.4,869,839	1.061478
Sprint performance	slope	0.1,886,897	0.2,517,766	0.75	0.459	−0.3,241,626	0.7,015,419
	bias	−0.8,569,031	0.6,525,468	−1.31	0.198	−2.186,097	0.4,722,912
Maximal force	slope	−1.151,145	0.8,730,931	−1.32	0.207	−3.012099	0.7,098,085
	bias	2.958,214	2.015052	1.47	0.163	−1.336,767	7.253,194
Change of direction ability	slope	0.4,479,726	0.2,743,476	1.63	0.113	−0.111,563	1.007508
	bias	−1.730,046	0.7,139,021	−2.42	0.021	−3.186,059	−0.2,740,326
Balance performance	slope	−6.810,937	2.130,842	−3.20	0.008	−11.45364	−2.168,231
	bias	13.8521	4.223,558	3.28	0.007	4.649,762	23.05445

## 4 Discussion

### 4.1 Maximal force

Maximal force, also known as absolute force, is the basis for the development of explosive power (Komi, 2003). Factors that influence maximal force are: muscle fiber type, neuromuscular factors, the size of the muscle cross-sectional area, and the level of relevant hormones in the body (Maijiu and Daqing, 2000). The results of this meta-analysis showed that UNI training improved the maximal force of the athletes’ unilateral limbs and BI training improved the maximal force of bilateral limbs. Further combing the literature found that it may be related to the movement pattern, and the training movements are significant to enhance the same movement pattern (Stern et al., 2020), the main reason is that the unilateral limb movement pattern needs to recruit more muscle groups than the bilateral limb movement pattern (Long, 2021), unilateral training can stimulate the human nervous system and more muscle fibers involved in contraction, especially the excitation intensity and number of fast muscle fibers increase, this stimulation is conducive to increase the contraction force of the muscle (Long, 2021). Pescatello et al. compared the training of only one limb with the training of no limb. Unilateral training is likely to rely on other redundant signal mechanisms for synthetic stimulation or on neural factors to regulate muscle strength and change (Pescatello et al., 2006). Wilkinson et al. found that unilateral training did not result in a significant increase in major synthetic and catabolic hormones induced by exercise, but was able to induce muscle hypertrophy and strength increases (Wilkinson et al., 2006). Migiano, et al. (Migiano et al., 2010) compared the endocrine response in the upper extremity immediately after unilateral and bilateral strength training and found no significant difference in circulating testosterone concentrations after unilateral and bilateral resistance training. Studies have shown that unilateral training can produce greater strength gain than bilateral training (Howard and Enoka, 1991; Botton et al., 2013), For people with bilateral limb strength imbalances, unilateral strength training can better compensate for the lack of bilateral strength and can also strengthen the weak limb, thus improving bilateral limb balance and reducing sports injuries. Jones M, et al. (Jones et al., 2012) studied the endocrine response of unilateral and bilateral resistance training, and found that before 30 min of resistance training, there was no significant difference in testosterone concentration between the two training cycles, but after 30 min of unilateral training, cortisol concentration decreased sharply. The concentration of immune reactive growth hormone, blood lactic acid and insulin was also lower than that of bilateral training. The results showed that the endocrine signals produced by different training modes were different, and the potential mechanism of muscle hypertrophy adaptation might also be different due to the difference of endocrine response. Hefzy, et al. (Hefzy et al., 1997) found that when performing anterior lunge exercises with a knee angle of 100°, 75% of the load was applied to the front leg, and they concluded that lunge exercises were superior to double leg squat exercises in improving lower limb strength.

Thus, the underlying mechanisms of strength and muscle hypertrophy adaptation may differ between unilateral and bilateral training in physiological expression due to differences in endocrine responses. Although the mechanisms that produce this difference between unilateral and bilateral training are unclear, a more consistent explanation is that there is a limitation of muscle neural activity in bilateral training that affects the maximization of muscle activation and the generation of maximal force (Ohtsuki, 1983; Vandervoort et al., 1984) and has been well documented in cross-sectional studies of different muscle groups (Taniguchi, 1997), populations (Kuruganti and Seaman, 2006) and test conditions.

### 4.2 Jumping performance

Jumping ability is the body through the central system of the brain regulation and control, through the body joints, muscles and ligaments and other coordination with each other to achieve the best state, the maximum explosive force of the lower limb muscle groups, so as to achieve the best jumping effect of technical action (Rutherford and jones, 1986). The results of this meta-analysis showed that unilateral training had a more significant effect on jumping ability in unilateral power model and bilateral training had a more significant effect on jumping ability in bilateral power model (Potter., 2017; Stern et al., 2020; Fahui, 2021). According to the specific training principle, unilateral training and bilateral training can improve the neuromuscular control ability of unilateral and bilateral movements respectively, and improve the performance of movements by increasing the number of motor units recruited, the release frequency of nerve impulses and coordination. Unilateral training can produce obvious neuromuscular adaptation effect on unilateral limbs. Bilateral training also showed positive effects on bilateral movements (Zhaoqing, 2021). It has been shown that unilateral training can improve jumping ability in bilateral power patterns (Boyle, 2010; Gonzalo-Skok et al., 2017a; Ye and Wangcheng, 2018; Yan and Hao, 2019; Yilin, 2020), that unilateral training reduces bilateral imbalance (Kobayashi et al., 2010), and that when imbalance is reduced on both sides, there may be a facilitative effect on jumping ability. When unilateral training is performed, muscle strength and neural activity also increase on the untrained side, a phenomenon known as the cross-migration effect (Howatson et al., 2013), and the increase in strength on the untrained side of the limb is accompanied by an increase in EMG activity, suggesting that central neural adaptation is the main driver of strength growth. The exact mechanism of cross-migration is unclear, but hypotheses have proposed that it may be due to neural adaptation, complex changes in contralateral motor pathways, and motor learning (Lee and Carroll, 2007). When cross-migration occurs, there is no significant increase in the cross-sectional area of the contralateral homologous muscle (Bezerra et al., 2009), and the cross-migration phenomenon may be based on the adaptation and regulation of the neuromuscular system by the cerebral cortex and spinal cord, which is weakly influenced by myogenic factors. Farthing, et al. (Farthing et al., 2007) performed 6 weeks of maximal isometric training on the training side and compared the level of homologous muscle activity on the non-training side before and after the intervention and noted that the level of activity in motor cortical areas and sensory cortical areas on the non-training side was significantly enhanced after the intervention. Tibor, et al. (Hortobagyi et al., 2009) stated that there is a close correlation between the level of motor cortex activity and the effect of training, and that unilateral training not only helps to enhance bilateral muscle strength, but also may reduce the inhibitory signals transmitted from the nervous centralis on the trained side to the nervous centralis on the untrained side. The cross-migration phenomenon relies mainly on the neuromodulation of the brain and spinal cord, Unilateral training first activates the central nerve on the non-training side, which is transmitted *via* the conduction pathway to the motor cortical area on the training side, while the motor cortical area on the non-training side is relatively inhibited, and the signal is transmitted *via* the corticospinal tract to the motor neurons in the anterior horn of the spinal cord, causing the spinal motor neurons to remain excited, thus affecting muscle contraction (Xiuhua et al., 2015).

### 4.3 Sprint performance

Speed is the shortest time it takes for the human body to complete a specific distance of movement (Miller, 2012). A meta-analysis confirmed that the increase in maximal force positively influenced the short-distance sprint speed (Comfort et al., 2012; Seitz et al., 2014). Comfort, et al. concluded that athletes with greater lower body strength would produce better sprint performance (Comfort et al., 2014). The results of this meta-analysis showed that unilateral training was more likely to improve the athletes’ straight-line sprinting ability. Derricks speris, et al. (Speirs et al., 2016) argued that the unilateral nature of sprinting is more suited to the biomechanical characteristics of unilateral training and that, at least from a kinematic perspective, sprinting is superficially less similar to bilateral training. The main factor affecting sprint ability is the pedal extension speed of lower limbs. To improve the pedal extension speed of lower limbs, it is necessary to develop the strength of muscle groups of lower limbs, including gluteus muscle, quadriceps muscle group of lower limbs, hamstring muscle group, triceps calf muscle and a series of small muscle groups involved in stabilizing and generating power around ankle joints (Lei, 2014). Unilateral training can promote strength growth in small and deep muscle groups, and this growth is precisely through nerve stimulation of the muscles, indicating that unilateral training helps to improve the nervous system’s ability to control the muscles and coordinate the strength of the upper and lower limbs during running. The nerve is also able to control the anterior tilt angle of the hip joint of the limb, thus improving the sensation of limb movement and the direction of onset of pushing away (Long, 2021). In the running process there will be a single-leg support phase, when you need to control the stability of the body and the ability to coordinate with the body, in order to reduce the sway of the body’s center of gravity, which is conducive to the speed of running. Therefore, unilateral training can effectively promote the athletes’ sprinting ability. In terms of training content arrangement, emphasis should be placed on Plyometric training (Stretching-shortening cycle), complex training, *etc.*, To enhance the vertical and horizontal ground reaction force, so as to improve the economy of running effect.

### 4.4 Change of direction ability

The ability to change direction is a multifactorial skill whose performance depends on neuromuscular coordination (Brughelli et al., 2008), leg muscle strength (Young et al., 2002; Sheppard and Young, 2006; Brughelli et al., 2008), and straight-line running speed (Young et al., 2002; Little and Williams, 2005; Castillo-Rodríguez et al., 2012). In the process of changing direction, the angle of cut and the approach speed before cut can influence the characteristics of knee joint loading, kinetic characteristics, kinematics, ground reaction force, muscle activation level, center of gravity velocity change, deceleration and force level, and technical action (Santos et al., 2018). The Angle of change direction and the approaching speed before cut-ins are the important factors that affect the mechanical characteristics of change motion. The results of this meta-analysis showed that there was no significant effect of unilateral and bilateral training on the indicators of the athletes’ ability to change direction. This is consistent with the findings of Yilin Que, in which she suggested that the reason for this result may be that the ability to change direction requires a single leg to be involved as a support foot for propulsion after a change of direction, which would to some extent affect the multidirectional speed performance after bilateral strength training intervention, and bilateral deficiency may be one of the reasons, i.e., the sports performance of the bilateral limbs is lower than the sum of the sports performance of the unilateral limbs (Yilin, 2020).

Although the results of this meta approached a significant effect (*p* = 0.06). However, the unilateral training enhancement was more significant. Stern, et al. (Stern et al., 2020) By comparing the effects of unilateral complex training and bilateral complex training on soccer players’ change of direction speed, it was found that both unilateral complex training and bilateral complex training improved change of direction speed to different degrees, with the unilateral group improving more significantly. The unilateral training movements are similar to the movement patterns of the change of direction ability techniques (deceleration pace, planting step reorganization, and propulsion pace) (Yilin, 2020). Among them, the rear leg elevation split-leg squat in resistance training can develop the athletes’ stable coordination ability and facilitate the athletes to adjust their center of body weight to a new direction during the change of direction phase; while the SSC mechanism of Plyometric training has a good migration effect on the cushioning ability in the deceleration pace and the acceleration ability in the propulsion pace phase. Li Zhaoqing (Zhaoqing, 2021) suggested that unilateral training can produce positive neuromuscular adaptation effects and lay the muscle strength foundation for improving the ability to change direction. According to the principle of specificity training, the more the human body performs centrifugal braking, change of direction and centripetal acceleration in vertical, horizontal front and back or lateral directions, the more similar the movement pattern is to the target task, and the greater the conversion effect of training, because unilateral training is closer to the mechanical characteristics of the change of direction movement. Mausehund, et al. (Mausehund et al., 2019) compared electromyographic information from barbell lunge, step-ups and rear elevated split squat and found that rear elevated split squat exhibited the highest level of hamstring activation (76% MVIC). They concluded that with the same relative load, the rear-foot elevated split-leg squat can better develop hamstring strength and contribute to the improvement of technical movements that require a lot of hamstring involvement such as landing brake and change of direction.

### 4.5 Balance performance

Human balance is one of the most important indicators of human physiological function. Balance is the body’s ability to coordinate stimuli from vestibular organs, muscles, tendons, receptors in joints and vision, which is the basic premise of human movement and maintaining posture and completing technical movements accurately (Peng, 2016). The results of this meta-analysis showed that unilateral and bilateral training had no significant effect on the balance ability of the athletes. A study by Shi Yan (Yan and Hao, 2019) confirmed that unilateral training significantly improved the Y balance test (YBT) of athletes. According to Michael Boyle, when training bilaterally, the body is in a relatively stable state and does not recruit more and deeper muscles to maintain body balance. However, when resistance training is performed, the improvement in limb muscle strength contributes to its balance, but the improvement is not significant. During unilateral training, the limbs are in an unstable state, and the intervention of force load is applied to force the hip joint and the body to produce confrontation and stability in the movement of the transverse section and frontal plane, so as to improve the spinal strength and maintain the stability and balance ability of the body (Boyle, 2010). In contrast, unilateral training can effectively improve the balance ability of athletes. By analyzing the characteristics of unilateral training, it was found that unilateral training can improve the balance ability of quadriceps (mainly rectus femoris, medial femoris and lateral femoris) and other muscle groups, but also has higher requirements for the stability of the ankle joint. Therefore, during training, emphasis should be placed on the development of proprioceptive control and innervated muscle capacity as a way to improve body balance and postural control. There are also studies to the contrary, where bilateral training is more effective for balance (Gonzalo-Skok et al., 2019). It is possible that the peak biceps femoris electromyographic (EMG) activity is greater with unilateral horizontal training than with bilateral vertical training, while the opposite is true for the lateral femoris in the upward phase (Murtagh et al., 2018). Thus, higher biceps femoris EMG and lateral femoris EMG may contribute to larger posterior medial and anterior medial distances, respectively, in the Star Excursion Balance Test (SEBT), and therefore it justifies the difference.

In conclusion, considering that the athletes have many years of training experience, in order to further improve the special competitive performance, the training content should be formulated for the special movement mechanics characteristics, energy supply system characteristics and muscle contraction forms, *etc.* Compared with the bilateral exercise content, unilateral exercises are more in line with the special training principles and can have a positive migration effect on sports performance. Unilateral training pattern (whether ground based or supported on an unstable base) can also provide an disruptive moment arm (torque) to the body, providing an additional means of increasing the core musculature (Behm et al., 2003). Exercises performed on unstable surfaces can not only increase core muscle activation, but can also increase limb muscle activation (Anderson and Behm, 2005; Marshall and Murphy, 2006a; Marshall and Murphy, 2006b) and co-contractions (Behm et al., 2002). However, other research demonstrates that ground-based lifts, such as squats and dead lifts, provide even higher core activation than callisthenic-style exercises performed on unstable surfaces (Hamlyn et al., 2007). Furthermore, unstable resisted actions can result in decreased force (Behm et al., 2002; Anderson and Behm, 2004; McBride et al., 2006), power (Cresswell and Thorstensson, 1994; Drinkwater et al., 2007), velocity, and range of motion (Drinkwater et al., 2007). Resistance trained individuals with years of experience performing ground-based free-weight lifts may not respond with higher activation of the core musculature when performing exercises on moderately unstable bases (Wahl and Behm, 2008).

Unilateral training and bilateral training both have similar neuromuscular control, and due to the specificity of unilateral training and the lower absolute load of unilateral training, it can reduce the sports injury brought by overuse. However, it is worth noting that as unilateral strength increases, the gradual increase in load based on unilateral support may lead to changes in movement technique, such as increased trunk flexion and rotation, increased pelvic tilt, and hip flexion and pronation (Costa et al., 2015; Eliassen et al., 2018; Anders et al., 2020). In addition, the unstable support points of unilateral training have the potential to limit the strength development of individuals in training and the magnitude of external loads applied to subsequently improve athletic performance (Argus et al., 2011). Therefore, unilateral training needs to be performed with technical proficiency in the movement and can be used as a supplement and aid to bilateral training.

### 4.6 Moderating variables

Unilateral and bilateral training with different intervention periods and intervention frequencies will also differ on motor performance, with intervention periods (≥8 weeks) and intervention frequencies (>2 sessions/week) improving maximal strength, change of direction ability and balance. A study of 16 weeks of high intensity strength training found that the first 8 weeks of training would increase maximal neural activation, the degree of muscle hypertrophy during this period would be insignificant, and after 8 weeks maximal neural activation would decrease and muscle hypertrophy would increase significantly. Therefore, for the maintenance of strength and muscle mass at 8–10 weeks is most appropriate, a 4–6 weeks training intervention would be too short, and a 13 weeks training intervention would lead to increased inertia of the organism (Jinao et al., 2011). This reinforces a widely accepted principle that prolonged training induces significant adaptations (Moran et al., 2018) that will increase the volume, strength, and explosive power of the trained muscle tissue. The increased concentration of anabolic hormones when training is performed is a signal of enhanced interaction between various target tissues, including skeletal muscle and hormones. When exercise stimulates motor units, various signals (electrical, chemical and hormonal information) are sent from the brain and active muscles to multiple endocrine glands (Haff and Triplett, 2016). Physiological systems, including the endocrine system, are very sensitive to the needs of active muscles, so the type of training program will determine the level of involvement of specific systems. Due to the unique stimulation of the nervous system induced by training, changes in the concentration of some hormones occur simultaneously in order to meet the demands of acute training volume, recovery and adaptation to training stress. Patterns of stress and hormonal responses are combined to produce an adaptive response of the tissue to a specific training program (Haff and Triplett, 2016). The magnitude of the hormonal response (i.e., anabolic or catabolic) depends on the amount of tissue stimulated, the amount of tissue remodeling, and the amount of tissue that needs to undergo repair due to exercise stress (Haff and Triplett., 2016). Therefore, the characteristics of the training stimulus (i.e., the choice of acute variables in the training program) are crucial for the hormonal response in the training program (Jones et al., 2012; Houde, 2021).

Complex training is a combination of resistance training and plyometric training, which provides a more comprehensive adaptation compared to single resistance training and plyometric training (Fatouros et al., 2000; Lee et al., 2014; Fathi et al., 2019; Zghal et al., 2019). Resistance training in complex training provides effective stimulation and activation of the nervous and muscular systems, allowing the individual to produce greater explosive power in the subsequent plyometric training (Ebben and Watts, 1998). In terms of exercise physiology, complex training increases motor unit excitability, which causes an increase in motor unit recruitment levels. Moreover, complex training modulates myosin light chain phosphorylation. Since myofilaments are overly sensitive to calcium ions and are again able to reduce presynaptic inhibition, they can provide conditions to enhance subsequent explosive output (Hodgson et al., 2005). Therefore, complex training is more able to stimulate and increase the excitability of the nervous system, thus improving maximal force and explosive power. The results of a meta-analysis by Pagaduan, et al. showed that complex training improved jumping ability more than plyometric training (Pagaduan and Pojskic, 2020). Numerous studies in the literature have confirmed that unilateral complex training is effective in improving athletic performance in athletes (Boyle, 2010; Yinlin, 2010; Stern et al., 2020; Fahui, 2021). Unilateral complex training is a training modality that combines unilateral resistance training with plyometric training, and its theoretical core basis is the same as plyometric training, which is the Stretch-Shortening Cycle (SSC), a SSC model that combines mechanical and neurophysiological mechanisms in which the muscle first undergoes rapid centrifugal elongation to activate the pull reflex and store elastic potential energy, allowing the subsequent centripetal contraction to exploit the principle that elastic energy is stored and re-released in the muscle and the force exploded by reflex recruitment of nerves is more powerful (Xiong and Zhaoji, 2014). Bilateral resistance training is more likely to improve jumping ability and balance. The reason for this is that resistance training has the strongest stimulus for skeletal muscle among many exercise modalities and has the effect of activating skeletal muscle protein synthesis, promoting muscle fiber hypertrophy, and improving muscle endurance and explosive power (Damas et al., 2018; Gallo-Villegas J et al., 2018; Grgic et al., 2018). J. Kraemer (Kraemer and Fleck, 2004) described the use of resistance training to increase explosive strength and skeletal muscle volume through the progress of research on resistance training, the design of training programs, and the relationship between muscle strength and local muscle endurance, muscle fiber and volume size. Resistance training also promotes positive balance in skeletal muscle protein metabolism levels, improves skeletal muscle strength and mass, increases class I and II fiber volume, and improves skeletal muscle explosive power and physical motility (Xiong and Zhaoji, 2014). In bilateral resistance training, the limb support range is larger and is in a relatively stable state, so there is no need to recruit more and deeper muscles to maintain body balance. Instead, after resistance training, the change in limb muscle strength can improve muscle coordination and overall body control, thus promoting balance to a certain extent.

In conclusion, with regard to the duration of intervention, interventions lasting ≥8 weeks and >2 sessions/week are more effective. In terms of interventions, emphasis should be placed on complex training, which can utilize the mechanical and neural efficacy of large load excitation to enhance the output power of subsequent plyometric training, improve neural excitability, reduce central delayed shelving, and contribute to increased force and power output during the centripetal contraction phase. Of course, other factors may also be present and their associated research evidence needs to be further explored in the future.

As mentioned earlier, there is an existing literature that empirically investigates the correlation between unilateral training and the acute endocrine response to exercise. Wilkinson et al. (Wilkinson et al., 2006) suggested that unilateral training induced muscle hypertrophy and increased strength, but did not result in a significant increase in major exercise-induced synthesis and breakdown hormones compared with bilateral training. Migiano et al. (Migiano et al., 2010) compared the immediate endocrine response after unilateral and bilateral upper limb strength training and found that there was no significant difference in circulating testosterone concentration between unilateral and bilateral resistance training. Some scholars chose to compare the training of only one limb with that of no training limb. UNI training is likely to rely on other redundant signal mechanisms to conduct synthetic stimulation or neural factors to adjust the strength and change of muscles (Pescatello et al., 2006). Therefore, the underlying mechanisms of strength and myohypertrophic adaptation at the level of gene expression may differ between UNI and BI training regimenes due to differences in endocrine responses.

Also in the neuromuscular context, it has been suggested that UNI training may produce greater strength gains than BI training (Howard and Enoka, 1991; Botton et al., 2013). It has been shown through electromyographic studies that unilateral plyometric training has a higher activation of the vastus medialis muscle, gastrocnemius muscle, and soleus muscle when using multi-joint exercise movements of the lower limbs and whole body (Soest et al., 1985), and unilateral resistance training has a higher activation of the obliquus externus abdominis, gluteus medius, and hamstrings, and a relatively lower activation of the rectus femoris, gluteus maximus, and erector spinae muscles (McCurdy et al., 2010; Saeterbakken and Fimland, 2012; Calatayud et al., 2015; Mausehund et al., 2019). It is evident that the muscles mobilized by UNI and BI are different; overall UNI stimulates the prime mover to a lesser extent and the fixator and synergistic muscle to a greater extent (Mudlo, 2014; Appleby et al., 2019), whereas unilateral explosive strength requires more efficient neuromuscular control for stability, and therefore unilateral training has a greater facilitation effect on unilateral explosive strength development (Fisher and Wallin, 2014). In summary, UNI and BI training both have similar neuromuscular control, but due to the specificity of UNI training and the smaller absolute load of a single session, motor injuries from overuse can be reduced. Therefore, UNI training can be used as a complementary and alternative method to BI training.

In addition to endocrine responses and neuromuscular adaptation changes, there is a unique physiological mechanism behind UNI training that supports this: the cross-training effect. Due to the significance of cross-training to the field of rehabilitation, several research paradigms have been published in the academic community on upper and lower extremity transfer effects, including muscle strength, skill learning, and bouncing movement learning. Meta-analyses by Carroll et al. (Carroll et al., 2006) and Manca et al. (Manca et al., 2017) predicted an effect of 8%–12% absolute gain in the contralateral limb after UNI strength training, or 52% of the strength gain in the trained limb. Of course these Meta-analyses did not include clinical cross-training studies (Dragert and Zehr, 2013; Magnus et al., 2013; Papandreou et al., 2013) or studies of UNI limb injuries (Magnus et al., 2010; Farthing et al., 2011). In these cases, the effect of cross-training is difficult to quantify because the strength parameters presented by the untrained limb are not only as to whether the strength of the untrained limb is increased, but also include the strength decay due to injury. To date, only 1 clinical fixation study of wrist fractures has been used to investigate cross-training as a standardized adjunctive treatment and to compare it with standard treatment alone (Magnus et al., 2013). A few studies have explored the value of cross-training in the application of maintaining muscle strength and size in the contralateral healthy limb in sports rehabilitation. Thus, UNI training is not only a methodological option for physical training, but also can be an adjunctive therapy for rehabilitation. Future research should focus more on the value of UNI training in the field of sports rehabilitation and the application of UNI training in the physical training of disabled athletes.

### 4.7 Limitations and future direction

There are some limitations to the current analysis, so our results should be interpreted with at least some caution. 1) The number of databases searched in the study was limited, and literature may have been missed. 2) Because of the diversity of UNI and BI training interventions, this study only explored resistance training, rapid stretch compound training, and compound training, and further subdivision of intervention types is needed in the future. 3) For inclusion in the study of the ability to influence balance, only dynamic balance was selected for analysis in this study due to the diversity of test evaluation methods, and future analysis of static balance is needed. 4) Coaches should reasonably arrange unilateral training time, because unilateral training takes twice as long as bilateral training, and too long training time will increase the athletes’ neural fatigue, thus reducing the training effect. Since the diagnostic tools used are different and the results obtained vary widely, it is suggested that future studies should focus on the duration, frequency and type of intervention of unilateral and bilateral training.

## 5 Conclusion

UNI training has a more significant effect on jumping ability and maximum strength for UNI power generation patterns, and BI training has a more significant effect on jumping ability and maximum strength for BI power generation patterns. UNI training is more specific to the specificity of the sport, especially for sports with unilateral limb dominant force (Stern et al., 2020). For change of direction and balance, UNI and BI training do not highlight that the training method is better, and the specific training plan should be developed according to the training objectives.
